# Prevalence of and Factors Associated with Myopia in Inner Mongolia Medical Students in China, a cross-sectional study

**DOI:** 10.1186/s12886-017-0446-y

**Published:** 2017-04-24

**Authors:** Lan Wang, Maolin Du, He Yi, Shengyun Duan, Wenfang Guo, Peng Qin, Zhihui Hao, Juan Sun

**Affiliations:** 1 Inner Mongolia Medical University, No. 5, Xinhua Street, Hohhot, Inner Mongolia Autonomous Region 010110 China; 2 Hohhot University for Nationalities, Hohhot, Inner Mongolia China; 3 Inner Mongolia People’s Hospital, Hohhot, Inner Mongolia China

**Keywords:** Myopia, Prevalence, Risk factors, Parent myopic, Myopia occurrence time

## Abstract

**Background:**

To further explore characteristics of myopia and changes in factors associated with myopia among students at Inner Mongolia Medical University.

**Methods:**

Two cross-sectional censuses were conducted in 2011 and 2013. Participants were medical students residing on campus in 2011 and 2013. Logistic regression analysis was performed to ascertain associations with basic information, genetic factors, environmental factors. The χ^2^ test was used to test for differences in prevalence between 2011 and 2013. Prevalence was calculated at various myopia occurrence times among different parental myopia statuses.

**Results:**

A total of 11,138 students enrolled from 2007 to 2012 completed the questionnaire. The prevalence of myopia in 2011 and 2013 was 70.50% and 69.21%, respectively, no statistically significant difference existed between the two censuses (*p* = 0.12). Both censuses were completed by 1015 students. There were no differences among the various year of study in 2011 or 2013. Myopic prevalence increased with an increased number of myopic parents: the prevalence if both parents were myopic was over 90%, nearly 80% if one parent was myopic, and less than 70% with non-myopic parents (*p* < 0.001). Myopic occurrence ranked from earliest to latest was in kindergarten and primary school when both parents were myopic, in middle school when one parent was myopic, and in university when no parent was myopic. Students staying up late, using a computer more than 3 h per day, not performing eye exercises, using eye drops, and rubbing the eyes at high risk for myopia.

**Conclusions:**

Myopic status was stable during the university period. Genetic factors play a major role in myopia. Protective measures are useful for university students.

## Background

Myopia is a significant public health problem and its prevalence is increasing over time [1]. By the year 2020, it is estimated that 2.5 billion people – one-third of the world’s population – will be affected by myopia [2]. Furthermore, the prevalence of myopia has been shown to vary widely with geographic location^3^. In European and North American adult populations, the prevalence of myopia is reported to be between 20% and 40% [3, 4]. In Asia, the prevalence of myopia among teenagers and young adults exceeds 70% [5, 6].

The etiology of myopia is multifactorial and both genes and the environment play important roles [7, 8]; myopia results from complex genetics [2, 9–12]. It has been shown that in young adults, education appear to cause increases in axial length and shifts toward increased myopia [13]. The high prevalence of myopia and high numbers of myopic university students pose particularly important public health and social problems [14]. Ocular risks associated with myopia should not be underestimated, and there is a public health need to prevent myopia onset and progression.

Based on the above research, there have been numerous studies both on myopia prevalence and associated factors. Therefore, we used a large sample to confirm the prevalence of myopia and factors associated with myopia among all students at an Inner Mongolian medical university.

## Methods

### Data source

Cross-sectional censuses of the physical and mental health of university students were conducted in 2011(6044) and 2013(6109) among medical students residing on campus at the Inner Mongolia Medical College of China. The censuses included students enrolled from 2007 to 2012, covering 6 years. Some students (1015) resided on campus in 2011 and 2013; therefore, they participated in both censuses. The total number of students is 11,138. In our school system, some students reside at school for 3 years, and others reside at school for 4 years [15]; therefore, we conducted the census twice. The two censuses employed a self-administered questionnaire. To make data expression clearer, we defined the factors newly appear in this paper, except those which had been defined in our previous studies [11, 15–17]. We also conducted a pre-survey to determine whether each factor could be accurately understood by the students and the factors which are easy to confound the data were modified. We also explained these factors in detail to the students in our census. The test-retest reliability was 0.96, which was calculated through a randomly sampled 100 students in the census [15].

### Census contents

Subjects evaluated as myopic were those who used myopic spectacles or contact lenses to look at objects and gave details about the age at which they started to wear spectacles or contact lenses [18].

We investigated factors including basic information: area (urban/rural), year of study (1, 2, 3, 4 and 5), and sex (male/female); genetic factors: family members’ myopic statuses (both parents, one parent, or no parent); and environmental factors: if they frequently see green (yes/no), perform eye exercises (insist on performing/ sometimes /rarely/ will not perform), use eye drops (yes/no), have an inadequate diet (yes/no), take breaks after reading 1 h (yes/no), use a lamp (yes/no), stay up late (yes/no), are affected by people around them staying up late (yes/no), stay up late for homework (yes/no), stay up late for study section review (yes/no), stay up late because of pressure to study (yes/no), search for information online (yes/no), when they started using a computer (primary school, high school, university), how often they used a computer (every day, 2–4 times a week, 1 time per week, almost none), how long they used a computer per day (less than 1 h, 1–3 h, more than 3 h), bedtime (before 22:00, 22:00–00:00, after 00:00, no regular time), read for long durations (yes/no), read while lying down (yes/no), read under dim light (yes/no), suffer from depression (yes/no), and if they rubbed their eyes (yes/no).

The dim light was lighting levels be below 30 footcandles (incandescent light bulbs below 40w) where the students usually reads and writes [19]. Participants excluded from the analyses included those reporting a history of cataract and/or glaucoma.

### Statistical analysis

The chi-squared test was used to test for differences in myopic prevalence between 2011 and 2013 in relation to various parameters. Because there was no significant difference in myopia prevalence between 2011 and 2013, we explored factors related to myopia prevalence by merging the two censuses. Prevalence was calculated for each investigated factor and various myopia occurrence times among students according to different parental myopia statuses. Multiple-factor non-conditional logistic regression analysis was used to evaluate the significance of each factor of myopia after adjusted for possible confounding factors. Dependent variables fell into two categories: myopic and non-myopic. Independent variables on the dependent variable in the model included all investigated factors. The odds ratio (OR) and corresponding 95% CI were calculated. In the model, ORs >1.0 designated increased myopic risk and ORs <1.0 indicated protective factors.

Microsoft Excel and SPSS 13.0 statistical software were used for data management and analysis. A statistical significance level of *p* ≤ 0.05 was used throughout the study.

## Results

A total of 11,138 students enrolled from 2007 to 2012 completed the questionnaire, of whom 7980 (27.3%) were men and 3149 (72.7%) were women. The mean age of the participants was 21.08 ± 1.57. The prevalence of myopia in 2011 and 2013 was 70.50 and 69.21, respectively, and no statistically significant difference existed between the two censuses (χ^2^ = 2.4, *p* = 0.12). One thousand fifteen students participated in both censuses, in which 694 myopic students were assessed in 2011, and only four myopic students were added in 2013.

Table 1 shows the baseline characteristics of the study participants and prevalence of myopia in relation to each census item. There was no difference among the various year of study in 2011 or 2013. Students’ myopic prevalence when both parents were myopic was over 90%; the prevalence when one parent was myopic was nearly 80%; and the prevalence when both parents were non-myopic was less than 70% (χ^2^ trend test = 18.23, *p* < 0.001). Myopic prevalence increased with an increased number of myopic parents according to the χ^2^ trend test. The prevalence of myopia was higher among women living in the city. The prevalence of myopia was also higher among students with staying up late, using a computer, lack of concern for eye health, lying down while reading, reading for a long duration, and going to bed after 10:00.

**Table 1:** Prevalence of myopia among Inner Mongolia Medical University students in relation to various parameters

Variable	*N* = 11,138	*n* = 7814	prevalence	χ^2^	P
Basic Information					
Sex				77.91	0.000
female	7980	5792	72.58		
male	3149	2018	64.08		
Area				76.89	0.000
Rural	6877	4622	67.21		
Urban	4239	3181	75.04		
Year of study				7.35	0.118
1	4276	2990	69.93		
2	3935	2755	70.01		
3	2260	1615	71.46		
4	513	361	70.37		
5	142	87	61.27		
Genetic factors					
Family members’ myopia statuses				18.23	0.000
both parents	245	221	90.20		
one parent	1448	1138	78.59		
no parent	9445	6455	68.34		
Environmental factors					
Often see green				0.73	0.390
No	4221	368	8.72		
Yes	6915	636	9.20		
Perform eye exercises				74.83	0.000
Insist on performing	325	181	55.69		
Sometimes	3581	2399	66.99		
Rarely	6522	4755	72.91		
Will not perform	699	475	67.95		
Eye drops				38.66	0.000
No	5993	4055	67.66		
Yes	5143	3758	73.07		
People around them stay up late				4.15	0.042
No	6957	4833	69.47		
Yes	4177	2978	71.30		
Inadequate diet				8.67	0.003
No	4748	3261	68.68		
Yes	6382	4548	71.26		
Take a break after reading 1 h				165.41	0.000
No	8722	6375	73.09		
Yes	2416	1439	59.56		
Use a lamp				103.54	0.000
No	2224	1364	61.33		
Yes	8913	6450	72.37		
Habit of staying up late				30.95	0.000
No	5330	3607	67.67		
Yes	5797	4203	72.50		
Stay up late for homework				9.61	0.002
No	5398	3712	68.77		
Yes	5735	4098	71.46		
Stay up late for study section review				1.67	0.195
No	7869	5492	69.79		
Yes	3265	2319	71.03		
Stay up late because of pressure to study				0.81	0.370
No	6023	4717	78.32		
Yes	5111	3640	71.22		
Search for information online				17.01	0.000
No	3200	2155	67.34		
Yes	7936	5658	71.30		
When they started using a computer				3.48	0.180
Primary school	1118	804	71.91		
High school	5470	3860	70.57		
University	4532	3143	69.35		
Frequency of computer use				3.1	0.380
Every day	3359	2344	69.78		
2–4 times a week	4025	2814	69.91		
1 time per week	2206	1544	69.99		
Almost none	1537	1108	72.09		
Computer use per day				4.17	0.125
Less than 1 h	3270	2338	71.50		
1–3 h	6098	4259	69.84		
More than 3 h	1753	1210	69.02		
Bedtime				31.04	0.000
Before 10:00	283	157	55.48		
10:00–12:00	8156	5776	70.82		
After 12:00	1961	1367	69.71		
No rule	730	511	70.00		
Read for long durations				1586.92	0.000
No	5906	3250	55.03		
Yes	5086	4557	89.60		
Read while lying down				9.26	0.002
No	2246	1517	67.54		
Yes	8886	6294	70.83		
Read under dim light				3.84	0.050
No	2374	1627	68.53		
Yes	8758	6184	70.61		
Depression				8.32	0.004
No	5270	3628	68.84		
Yes	5860	4181	71.35		
Eye rubbing				19.04	0.000
No	2752	1840	66.86		
Yes	8379	5970	71.25		

Table 2 shows the myopia statuses of the students. The results suggest that nearly 80% began wearing spectacles in middle school. Regarding the type of glasses, more than 80% wore frame glasses and more than half chose them in an eyeglasses store. Among myopic students, 53.44% envied normal vision and 56.91% felt eye fatigue. The attitude of 60% of myopic students was open to trying treatment.

**Table 2:** The myopia statuses of students at Inner Mongolia Medical University

Category	*n* = 7814	prevalence	χ^2^	p
When they began wearing spectacles			17,197.49	0.000
Kindergarten	54	0.69		
Primary school	687	8.79		
Middle school	6108	78.17		
University	965	12.35		
Where they were fitted for spectacles			6525.73	0.000
Ophthalmic hospital	2968	37.98		
Eyeglasses Store	5178	66.27		
Both	332	4.25		
Glasses Type				
Contact lenses	278	3.56	18,286.13	0.000
Frame glasses	6476	82.88		
Both	794	10.16		
Do not wear glasses	385	4.93		
Views on myopia			1983.44	0.000
Worried about genetics	2805	35.90		
Lack of confidence	2076	26.57		
Envy normal vision	4176	53.44		
Eye fatigue	4447	56.91		
Views on treating myopia			5201.18	0.000
Willing to try treatment	4681	59.91		
No need to be overly concerned	2815	36.03		
Do not care	427	5.46		

We included all factors in binary logistic regression models (Table 3). Students with one or two myopic parents were at high risk for myopia. Women who lived in the city with staying up late, using a computer more than 3 h per day, not performing eye exercises, using eye drops, rubbing their eyes were at high risk for myopia. Taking a break after reading for 1 h and not reading under a dim lamp were protective factors.

**Table 3:** Results of the logistic regression analysis on myopia among medical students

	P	OR	95% CI
Sex			
female		1	
male	0.000	0.64	0.58–0.71
Area			
Rural		1	
Urban	0.000	1.14	1.03–1.27
Family members’ myopia statuses			
father	0.000	1.71	1.41–2.08
mother	0.001	1.37	1.13–1.67
Take a break after reading 1 h			
No		1	
Yes	0.000	0.56	0.50–0.63
Reading under a dim lamp			
No		1	
Yes	0.000	1.47	1.31–1.64
Habit of staying up late			
No		1	
Yes	0.000	1.16	1.06–1.27
Perform eye exercises			
Insist on performing		1	
Sometimes	0.13	0.77	0.56–1.08
Rarely	0.72	1.04	0.84–1.28
Do not perform	0.01	1.29	1.05–1.57
Computer use per day			
Less than 1 h		1	
1–3 h	0.00	1.33	1.14–1.55
More than 3 h	0.03	1.16	1.01–1.34
Bedtime			
Before 10:00		1	
10:00–12:00	0.325	0.84	0.59–1.19
After 12:00	0.123	1.16	0.96–1.41
No rule	0.926	0.99	0.80–1.22
Often see green			
No			
Yes	0.001	1.17	1.06–1.29
Stay up to for search for information online			
No			
Yes	0.01	1.15	1.03–1.29
Eye rubbing			
No			
Yes	0.02	1.13	1.02–1.26
Eye drops			
No			
Yes	0.001	1.17	1.06–1.29

*CI* confidence interval

Table 4 shows the time at which myopia occurred among students with different parental myopia statuses. The occurrence time of student myopia was earliest in kindergarten and primary school when both parents were myopic. The occurrence time of student myopia ranked second in middle school when one parent was myopic. The occurrence time was latest in university when neither parent was myopic.

**Table 4:** Student myopia occurrence time among different parental myopia statuses

	father		mother		both myopic		both non-myopic	
Occurrence time	n	%	n	%	n	%	n	%
Kindergarten	5	0.82	2	0.38	2	0.90	45	0.70
Primary school	88	14.43	70	13.26	81	36.65	448	6.94
Middle school	501	82.13	437	82.77	129	58.37	5041	78.09
University	16	2.62	19	3.60	9	4.07	921	14.27
total	610	100.00	528	100.00	221	100.00	6455	100.00

## Discussion

Compared with the reported prevalence of myopia among the general population in Western countries, the prevalence of myopia in our study was considerably higher [20, 21]. Compared with medical students in other countries, the prevalence of myopia in our study was also higher [22, 23].

We performed two censuses of all students residing on the university campus in 2011 and 2013. There was no statistically significant difference between the two censuses in myopic prevalence. Further, the myopic prevalence of students who participated in both censuses was nearly unchanged. The prevalence of myopia was not significantly different from year of study 1 to year of study 5 in 2011 or 2013. The results suggest that myopic status was stable and did not significantly change during the university period. A study among university students was consistent with our result [14]. The results may be explained by genetic factors. Students’ myopic prevalence when both parents were myopic, when one parent was myopic, and when both parents were non-myopic showed a dose-dependent relationship. It showed that the majority of myopia cases within populations are caused by hereditary factors. In addition, the occurrence time of student myopia was the earliest in kindergarten and primary school when both parents were myopic. The occurrence time of student myopia was second earliest in middle school when one parent was myopic. Student myopia occurred latest in university when no parent was myopic. It clarified that student myopia occurs earlier with an increased number of myopic parents. Several studies have suggested relationships between heredity and myopia [2, 9]. Our results are consistent with their conclusions and confirmed that people were more likely to develop myopia earlier because of heredity from myopic parents [9].

While genetic factors play a major role, environmental factors also play a role in lens thickness changes, but do not change myopic status [12]. A previous study confirmed that environmental change causes myopia [12]. We further explored myopia-related environmental factors among 11,138 students. In our study, taking a break after reading 1 h and not studying under a dim lamp had protective effects on eye health. It clarified that a healthy lifestyle played a protective role in university students. On the contrary staying up late, using a computer more than 3 h per day, not performing eye exercises, using eye drops, and rubbing the eyes could increase the thickness of the glasses. Moreover, it was demonstrated that some protective measures were useful for medical university students and could prevent further increases in the thickness of their glasses.

## Conclusions

Myopic status was stable during the university period. Genetic factors play a major role in myopia. Taking a break after reading 1 h and not studying under a dim lamp had protective effects on eye health. Staying up late, using a computer more than 3 h per day, not performing eye exercises, using eye drops, and rubbing the eyes could increase the thickness of the glasses and all above can effectively change through education, so university administrators should provide systematic education to enhance it in university students.

### Limitation

In our study, we did not perform an eye examination for all students; thus, “myopic” was defined according to the individual student’s report that they “myopic used spectacles or contact lenses either occasionally or frequently” during our study. Therefore, the prevalence of myopia may be lower because some slightly myopic students may choose not to wear glasses.
